# Perioperative Use of Clevidipine: A Systematic Review and Meta-Analysis

**DOI:** 10.1371/journal.pone.0150625

**Published:** 2016-03-28

**Authors:** Angel Espinosa, Javier Ripollés–Melchor, Rubén Casans-Francés, Alfredo Abad-Gurumeta, Sergio D. Bergese, Alix Zuleta-Alarcon, Francisco López-Timoneda, José María Calvo-Vecino, Evidence Anesthesia Review Group

**Affiliations:** 1 Center of vascular and thoracic surgery and intensive care, Örebro University Hospital, Örebro, Sweden; 2 Department of Anesthesia, Complutense University of Madrid, Infanta Leonor University Hospital, Madrid, Spain; 3 Department of Anesthesia, University of Zaragoza, Lozano Blesa University Hospital Clinic, Zaragoza, Spain; 4 Department of Anesthesia, La Paz University Hospital, Madrid, Spain; 5 Departments of Anesthesiology and Neurological Surgery, The Ohio State University, Columbus, Ohio, United States of America; 6 Department of Anesthesia, Complutense University of Madrid, San Carlos University Hospital, Madrid, Spain; Kurume University School of Medicine, JAPAN

## Abstract

**Background:**

Clevidipine is an ultrashort-acting drug for rapid reduction of blood pressure by selectively acting on the L-type Ca2+ channels on arteriolar smooth muscle. The drug’s ultrashort action in reducing the blood pressure is due to its rapid hydrolysis by blood and extravascular tissue esterases, which does not depend on hepato-renal metabolism and excretion. An analysis of the perioperative management of blood pressure should be considered to compare with other intravenous antihypertensive agents.

**Methods:**

Analyses of the available evidence in randomized clinical trials following the PRISMA methodology as well as clinical significance according to the GRADE system were conducted. Placebo versus other antihypertensive drugs studies were included. Statistical assessments were done using the X2 and I2 tests.

**Results:**

Clevidipine was more effective in maintaining the blood pressure within pre-specified ranges compared with other antihypertensive drugs (MD, -17.87 CI 95%: -29.02 to -6.72; p = 0.02). The use of Clevidipine versus placebo and rescue antihypertensive intravenous drug showed a clear reduction in rates of treatment failure (RR 0.10; IC 95%; 0.05–0.18; p <0.0001). There was no difference in the incidence of adverse events compared with placebo (RR 1.47; 95% CI 0.89 to 2.43, p = 0.14) and with other antihypertensive drugs (RR 0.78, 95% CI 0.45 to 1.35; p = 0.37). In addition, there was no difference in the incidence of atrial fibrillation (AF) between clevidipine and control groups (RR 1.09, IC del 95%: 0.65 a 1.83; p = 0.73).

**Conclusions:**

Clevidipine is an ultrafast-acting drug that is highly effective for management of perioperative arterial hypertension. It is devoid of adverse effects associated with the use of other IV antihypertensives. Its favorable pharmacodynamic and pharmacokinetic properties make clevidipine the drug of choice for the management of acute perioperative hypertension. It is important to emphasize the need for further studies with a larger number of patients to confirm these findings and increase the degree of evidence.

## Introduction

Multiple intravenous medications are currently used to control blood pressure (BP) in the perioperative period, and all these drugs have advantages and disadvantages [1, 2].

Perioperative Blood Pressure (BP) in hypertensive patients has been associated with a worse outcome, hence, many treatment protocols require invasive BP monitoring during high-risk procedures [3]. Acute perioperative hypertension (HTA) affects up to 80% of patients undergoing cardiac surgeries and over 25% of patients undergoing major non-cardiac procedures [4].

Pre-existing hypertension (affecting more than two-thirds of cardio surgical patients) contributes to development of acute perioperative hypertension and often is a common reason for postponing surgery [5, 6].

Other ultrafast-acting drugs such as nitroglycerin or nitroprusside have the disadvantage of producing intense venodilation, which may decrease the preload and impair pulmonary circulation. These effects can offset the benefits of rapid BP control.

Others drugs like dihydralazine are effective in lowering BP by intravenous bolus but have a residual effect that can last for hours. Labetalol can generate undesired cardiovascular effects that will narrow its clinical utility. Urapidil is a commonly used perioperative drug with a dual mechanism of action: α1-adrenoceptor antagonism, 5-HT1A receptor agonism, and possible central α2-adrenoceptor agonism. Its use has been associated with postsurgical hypotension after 1 hour of continuous infusion [7].

Clevidipine Butyrate acts on L-type Ca^2+^ channels that regulate the Ca^2+^ flow in arteriolar smooth muscle cells during depolarization. By relaxing the arteriolar smooth muscle, it reduces peripheral vascular resistance, increases cardiac output and reduces blood pressure. Clevidipine has no effect on capacitance vessels and the venous tone, and it does not produce undesirable changes in afterload, including the left ventricle filling pressure and left ventricular peak pressure [8]. Clevidipine is a dihydropyridine just as nicardipine and nifedipine, which are considered first-line drugs for hypertensive emergencies owing to their strong vasodilatory effects and low propensity to cause abnormalities in cardiac conduction and contractility [9–11].

Several studies have shown clevidipine’s potential in blood pressure maintenance. A study published in in 2007 showed that preoperative clevidipine administration was effective in decreasing blood pressure and achieving a 92.5% rate of treatment success with a failure of 7.5% when compared with placebo (82.7%, 43 of 52; *P* _ 0.0001). The authors agreed that a “modest” increase in heart rate from baseline values was reported for the clevidipine group. However the study considers 105 patients for randomization with only 53 patients receiving clevidipine and 52 placebo.[12] One year later, in 2008, Singla et al. reported similar treatment success rate (91.8%) when Clevidipine was administrated in post operatory setting. The study analyzed data collected from only 69 patients dosed with study drug and 49 placebo recipients. [13] The ECLIPSE study results–published in 2008 –showed no difference in the incidence of myocardial infarction, stroke or renal dysfunction for patients treated with Clevidipine when compared with other antihypertensive drugs (nitroglycerin, sodium nitroprusside, and nicardipine).[14] This is an open label, perioperative study design, reporting outcome from 752 patients receiving clevidipine compared with 756 patients receiving different comparator drugs.[14]

The existing published data analyzed results obtained from studies investigating the outcome of clevidipine administration at different time points during perioperative intervention. Our study offers an integrated analysis of clevidipine administration during the pre-operative, intraoperative, and post-operative period.

In an attempt to assess the effectiveness of clevidipine as an optimal agent for perioperative blood pressure management, we analyzed the accumulated evidence of the intraoperative use of clevidipine in adults and compared its safety and efficacy on blood pressure management relative to other existing hypotensive drugs used during anesthesia.

## Material and Methods

### Study design

Systematic review according to the Preferred Reporting Items for Systematic Reviews and Meta- Analyzes (PRISMA) statements [15], The Cochrane Handbook for Systematic Reviews of Interventions [16] and Jakobsen et al. "8 steps methodological recommendations" [17].

The absence of sufficient data in studies prevented the protocol to be included in the PROSPERO registration, although P-PRISMA agreement has been faithfully followed (S1 PRISMA Checklist) [18].

### Inclusion criteria

PRISMA [15] methodology was used for selecting studies, based on the following criteria:

1. Participants: Adult patients scheduled for surgery in which Clevidipine was administered to control blood pressure in the perioperative period.
2. Type of Intervention: Administration Clevidipine at any dose.
3. Type of comparison: placebo or other antihypertensive drugs.
4. Types of studies: Randomized clinical trial (RCT) in which the effectiveness of antihypertensive therapy and/or complications associated with treatment and adverse effects associated with the drug are reported and analyzed. Those presented as posters or conference papers were excluded.

### Source of information

Different search strategies (last updated in January 2015) were established to identify relevant studies containing the inclusion criteria, using EMBASE, MEDLINE, and the Cochrane Library.

### Search items

Keywords such as “Clevidipine” and “randomized control trial” were used. Studies were restricted to adult human subjects. There was no restriction on date or language. RCTs with complete published data only were included. There was no limited period of time in the literature search.

### Study selection and data extraction

Two independent researchers assessed each title and abstract, to rule out any irrelevant RCT, and identify potentially relevant ones. Those who met the inclusion criteria described above were selected. The data extraction was performed by two different researchers, and a third investigator was required to answer any discrepancy by analyzing more in depth. The authors reviewed data analysis during the transcription process to avoid errors.

### Item data

PICOS characteristics (patient, intervention, comparison, outcome and design) of the included studies were collected.

On the other hand, data on the frequency, type of complications, and adverse events of related drugs were collected.

We used the PICO system to set the research question, with a universe of Patients including adults from 18 years old and older, Intervention being the use of an infusion of an antihypertensive medication with a Comparison of Clevidipine vs. placebo plus rescue antihypertensive intravenous drug or a control drug, and the Outcome the safety and efficacy of the blood pressure control between pre-established limits.

The question was formulated as n°1 –in patients with perioperative hypertension, is Clevidipine either more effective or safe for maintaining the blood pressure in a specific range than other antihypertensive drugs? And n°2 –in patients with perioperative hypertension, is Clevidipine either more effective or safe for maintaining the blood pressure in a specific range than placebo plus rescue antihypertensive intravenous drug?

### Bias assessment of the included studies

Cochrane test for assessment of possible bias was implemented [16]. We used seven domains of this test to evaluate the quality of methodologies of the studies included in the analysis. If one or more domains were determined to be high risk, we classified the RCT as having a high risk of bias. If the test cannot produce final results, the RCT was also classified as having a high risk for bias [16].

### Outcome endpoints

#### Effectiveness of Clevidipine vs. Placebo plus rescue antihypertensive intravenous drug

The incidence of treatment failure was defined as the inability to lower systolic blood pressure below 15% of the baseline or premature and permanent interruption of the trial treatment for any reason within 30 minutes after the onset of the drug. Alternative antihypertensive treatment could be instituted as per institutional practice after treatment failure.

#### Effectiveness of Clevidipine vs. other antihypertensive drugs

Evaluation using the analysis of the area under the curve (AUC) of the excursions of the blood pressure (BP) beyond the upper and lower limits defaults, normalized per hour (AUCSBP—D), or defined as the total area off the curve of time, mean arterial pressure (i.e. both above and below the clinical range predefined as a target), and normalized by time (AUCMAP-D, in units of mmHg · min ·hr-1).

#### Clevidipine safety compared to placebo plus rescue antihypertensive intravenous drug and to other antihypertensive drugs

The incidence of reported serious adverse events (as defined by the authors), including atrial fibrillation. Drug-related adverse events defined as the incidence of adverse events (AEs) since the beginning of the study drug and evaluated by a physician regardless of the relationship with Clevidipine.

### Statistical analysis

Review manager (“Revman 5.2.3”) [19] for MAC (Cochrane collaboration, Oxford, UK) and OpenMetanalyst [20] were used.

To measure the effect of dichotomous and continuous variables, risk ratio (RR) and mean difference was used respectively, both having a 95% confidence interval.

The model of Mantel-Haenszel for random effects was used as statistical method of the meta-analysis for the dichotomous variables, and inverse variance for continuous variables [21]; the results were presented as relative risk (RR) with 95% confidence interval.

Forest plot was built considering p < 0.05 as statistically significant effect. Statistical heterogeneity was assessed using the I2 statistic [22]; I2 less than 25 percent was defined as low heterogeneity; between 25 and 50 percent, moderate heterogeneity; greater than 50 percent, high heterogeneity. One χ2 test was conducted for heterogeneity, considering p value of < 0.10 as statistically significant.

When statistical heterogeneity was found, results of the meta-analysis were presented using a random effects model. When no statistical heterogeneity was found, the results were presented as fixed effects model. “Funnel plot” technique was used for the analysis of publication bias, only if at least ten clinical trials were included in the meta-analysis [16].

### Level of evidence and clinical significance

The GRADE [23] methodology was used to evaluate the quality of the evidence (www.gradeworkinggroup.org) of our findings. A thorough assessment of the balance between beneficial and harmful effects of Clevidipine was performed to evaluate the clinical significance of the effects of the intervention [24].

## Results

### Studies selection

**Fig 1:**
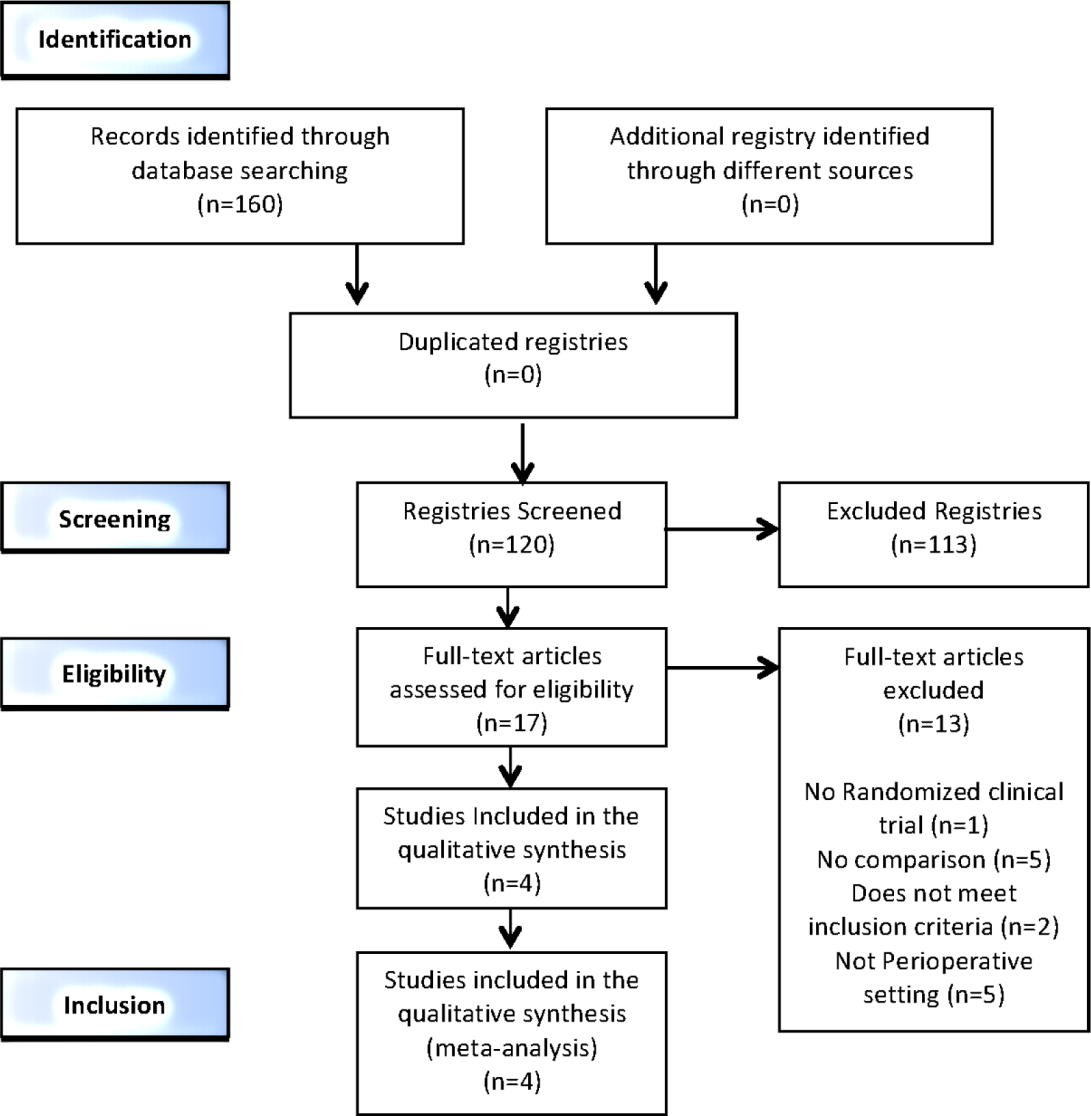
Prisma flow diagram. Flow diagram illustrating search strategy.

**Fig 2:**
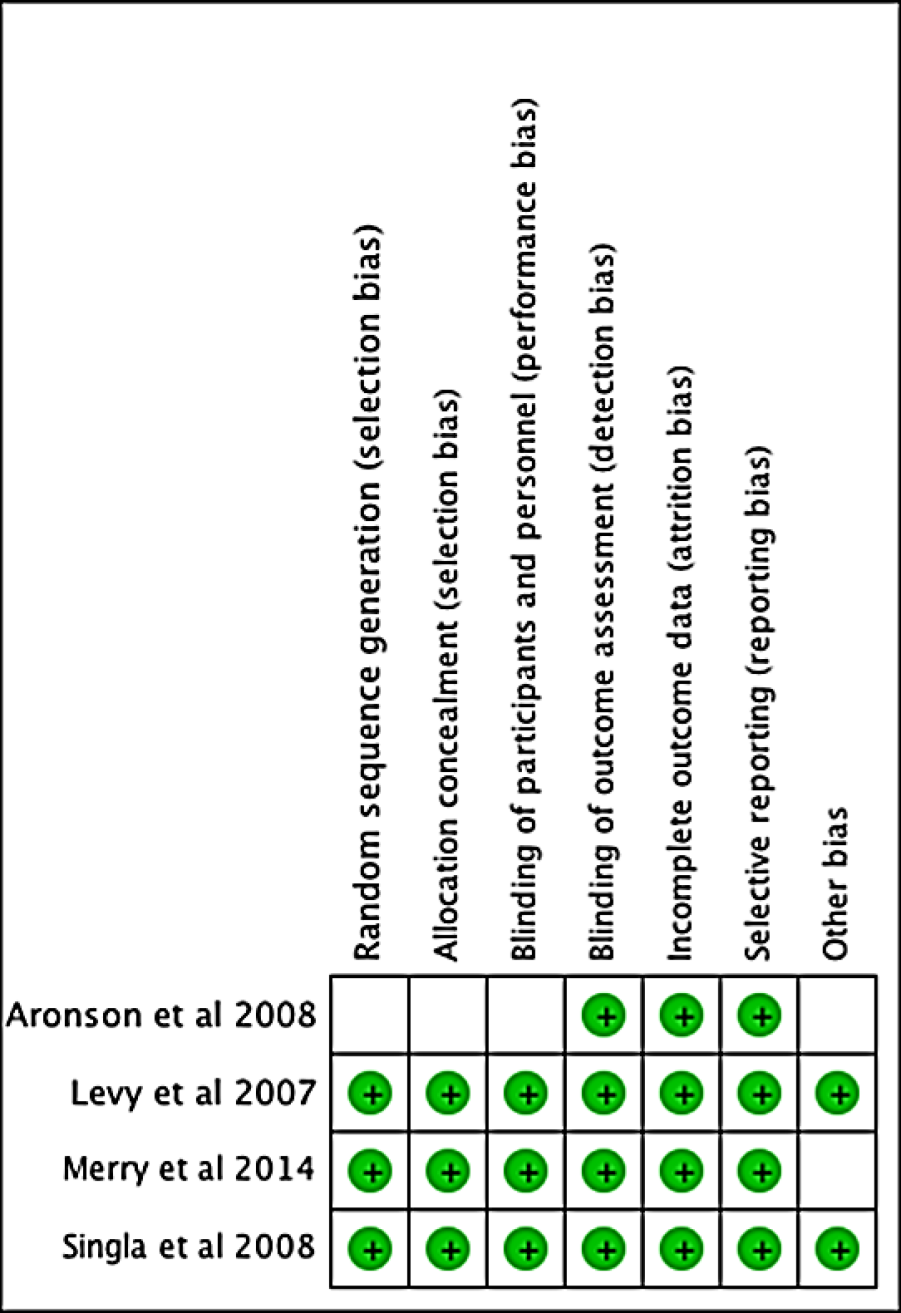
Risk of bias summary. Review authors' judgments about each risk of bias item for each included study.

Of the 160 references found in databases, 17 were fully analyzed, and finally, 4 were included for systematic review and meta-analysis (See Fig 1). Two independent reviewers analyzed the risk of bias in Cochrane tool [16]. Any disparity was resolved by involving a third reviewer. Methodological quality was presented in a summary table (Fig 2). RCTs that evaluated the use of Clevidipine versus placebo plus rescue antihypertensive intravenous drug or versus other antihypertensive drugs were selected to be included in the analysis. The results are presented in Tables 1, 2 and 3. (In addition, 1 article was not included in the analysis, due to inconsistencies on efficacy, although it is considered important and is shown on the table).

**Table 1:** PICO Characteristics of Included Studies.

Study	Year	Patients	N	Intervention	Comparison	Outcomes	Design	Country	Financed
**Powroznyk et al.**	2003	CABG intervened adult patients	39	Clevidipine 0,3mcg/kg/min	Nitroprusside 0,5mcg/kg/min	Blood pressure control. Hemodynamic parameters	Multicenter randomized clinical trial	UK	AstraZeneca R&D Möndal, Sweden
**Levy et al. (ESCAPE-1)**	2007	Cardiac surgery intervened adult patients	105	Clevidipine 0,4–8 mcg/kg/min	Placebo (rescue: Antihypertensive drug not specified)	Antihypertensive efficacy (Decrease in SBP >15% of baseline) during the first 30 minutes	Placebo-controlled multicenter randomized clinical trial	USA	The Medicines Company
**Singla et al. (ESCAPE-2)**	2008	Cardiac surgery intervened adult patients	110	Clevidipine 0,4–8 mcg/kg/min	Placebo (rescue: Antihypertensive drug not specified)	Antihypertensive efficacy (Decrease in SBP >15% of baseline) during the first 30 minutes	Placebo-controlled multicenter randomized clinical trial	USA	The Medicines Company
**Aronson et al. (ECLIPSE)**	2008	Cardiac surgery intervened adult patients	1507	Clevidipine 0,3–8mcg/kg/min	Nitroprusside, nitroglycerine or nicardipine	Safety assessed by the incidence of myocardial infarction, death, Stroke, renal dysfunction. Assessment of Clevidipine efficacy using the analysis of the area under the curve of blood pressure normalized per hour.	Open prospective multicenter randomized clinical trial	USA	Not stated
**Merry et al.**	2014	Cardiac surgery intervened adult patients	101	Clevidipine 0,2–8mcg/kg/min	Nitroglycerine 0,4mcg/kg/min to maximum dose	Assessment of Clevidipine efficacy using the analysis of the area under the curve of blood pressure	Multicenter randomized clinical trial. Not inferiority study	USA, New Zealand	The Medicines Company

**Table 2:** Complications.

Study	Year	Intervention group complications	Intervention group severe complications	Control group complications	Control group severe complications
**Powroznyk et al.**	2003	Not stated	Not stated	Not stated	Not stated
**Levy et al. (ESCAPE-1)**	2007	Fever 18.9%	Death as a consequence of mediastinal hemorrhage, not related with the study drug (1 patient 1.9%)	Fever 13.7%	Myocardial infarction 2,9%
		Atrial fibrillation 13.2%		Atrial fibrillation 11,8%	
		Acute renal dysfunction/failure 9.4%		Acute renal dysfunction/failure 2,0%	
		Nausea 5.7%		Nausea 9,8%	
**Singla et al. (ESCAPE-2)**	2008	Nausea 21,3%	Pneumonia, respiratory failure 3,3%	Nausea 12,2%	No stated
		Atrial fibrillation 21,3%	Postoperative hemorrhage 0,6%	Atrial fibrillation 12,2%,	
		Insomnia 11,5%		Insomnia 6,1%,	
		Edema 8,2%		Edema 12,2%	
		Atelectasis 3,3%		Atelectasis 10,2	
**Aronson et al (ECLIPSE)**	2008		Atrial fibrillation 2,4%		Atrial fibrillation 2,4%,
			Respiratory failure 1,1%		Respiratory failure 2,5%,
			Acute renal failure 2,3%		Acute renal failure1,7%,
			Ventricular fibrillation 0,9%		Ventricular fibrillation 1,5%,
			Cardiac arrest 0,5%		Cardiac arrest 1,1%,
			Stroke 0,5%		Stroke 1,1%,
			Postoperative hemorrhage 0,5%		Postoperative hemorrhage 1,1%
**Merry et al.**	2014	63.3%	6%	58.8%	Acute myocardial infarction 1%
			Acute myocardial infarction 4%		Atrial fibrillation 9.8%
			Atrial fibrillation 2%		

**Table 3:** Drug-related Adverse Events.

Study	Year	Adverse events reported with clevidipine (%)	Type of adverse event reported with clevidipine
**Powroznyk et al.**	2003	No	No
**Levy et al. (ESCAPE-1)**	2007	9,4%	No
**Singla et al (ESCAPE-2)**	2008	0,6%	Thrombophlebitis
**Aronson et al. (ECLIPSE)**	2008	0%	No
**Merry et al.**	2014	0%	No

### Primary results

#### Efficacy of Clevidipine vs Placebo plus rescue antihypertensive intravenous drug

**Fig 3:**
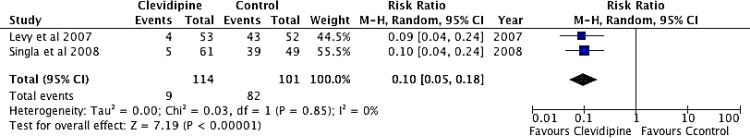
Forest Plot. Efficacy of Clevidipine vs Placebo. Forest plot was built considering p < 0.05 as statistically significant effect.

The use of Clevidipine vs placebo, significantly decreased the failure in treatment (RR 0.10; IC 95%; 0.05–0.18; p <0.0001) (Fig 3).

#### Efficacy of Clevidipine vs other antihypertensive drugs

**Fig 4:**
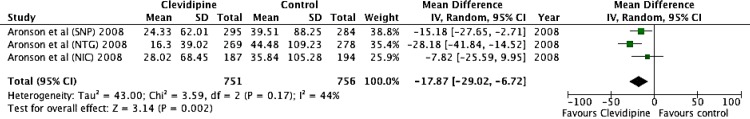
Forest Plot. Efficacy of Clevidipine vs other antihypertensive drugs. Forest plot considering p < 0.05 as statistically significant. NIC, denotes nicardipine; NTG, nitroglycerine; SNP, nitroprusside.

AUC SBP-D: Median AUC SBP-D was significantly lower in patients treated with Clevidipine than in patients treated with other antihypertensives (MD, -17.87 CI 95%: -29.02 to -6.72; p = 0.02) (Fig 4).

#### Safety

**Fig 5:**
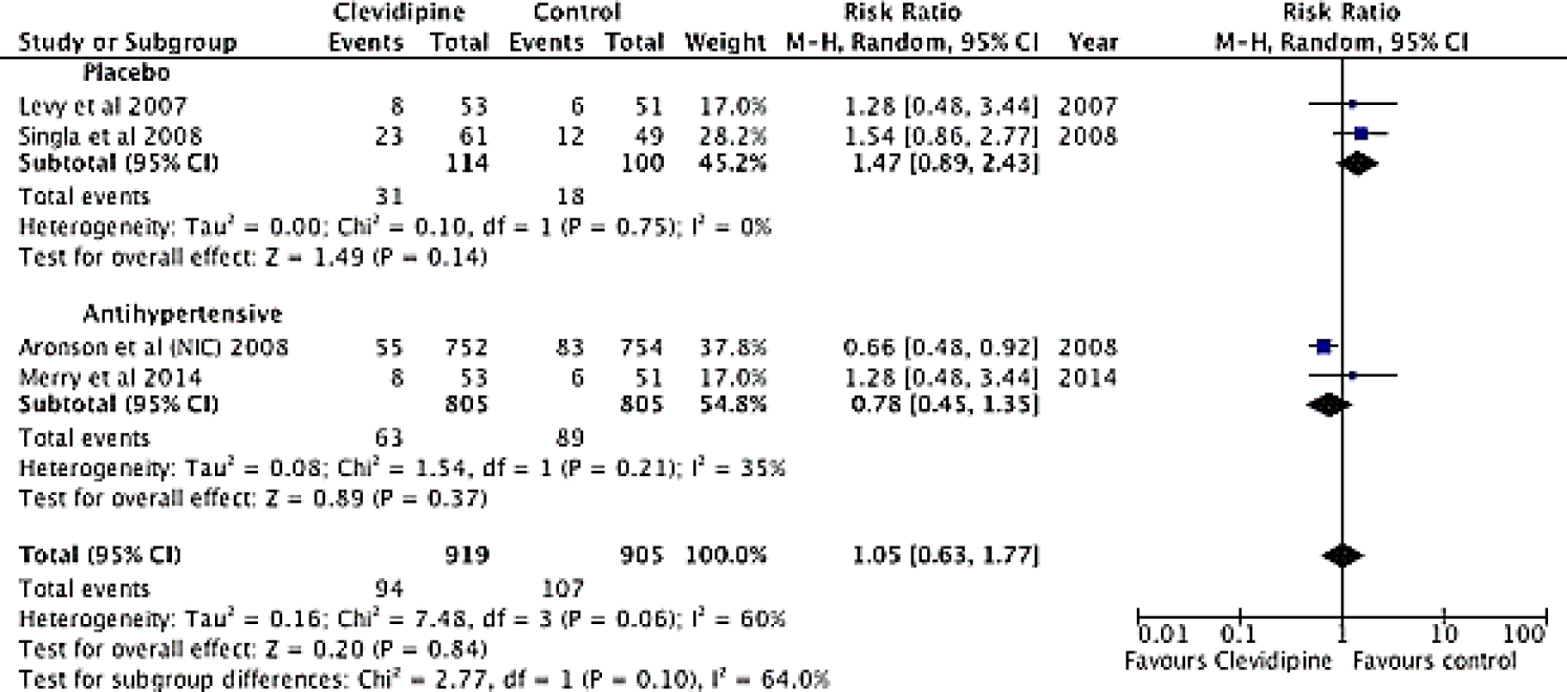
Forest Plot. Safety. Forest plot considering p < 0.05 as statistically significant. One χ2 test was conducted for heterogeneity, considering p value of < 0.10 as statistically significant.

Differences in the incidence of adverse events between Clevidipine and placebo and rescue antihypertensive intravenous drug groups (RR 1.47; 95% CI 0.89 to 2.43, p = 0.14), in the comparison with other antihypertensive drugs (RR 0.78, 95% CI 0.45 to 1.35; p = 0.37) or when analyzed together (RR 1.05; 95% CI: 0.63 to 1.77; p = 0.06) were not found (Fig 5).

#### Atrial fibrillation

**Fig 6:**
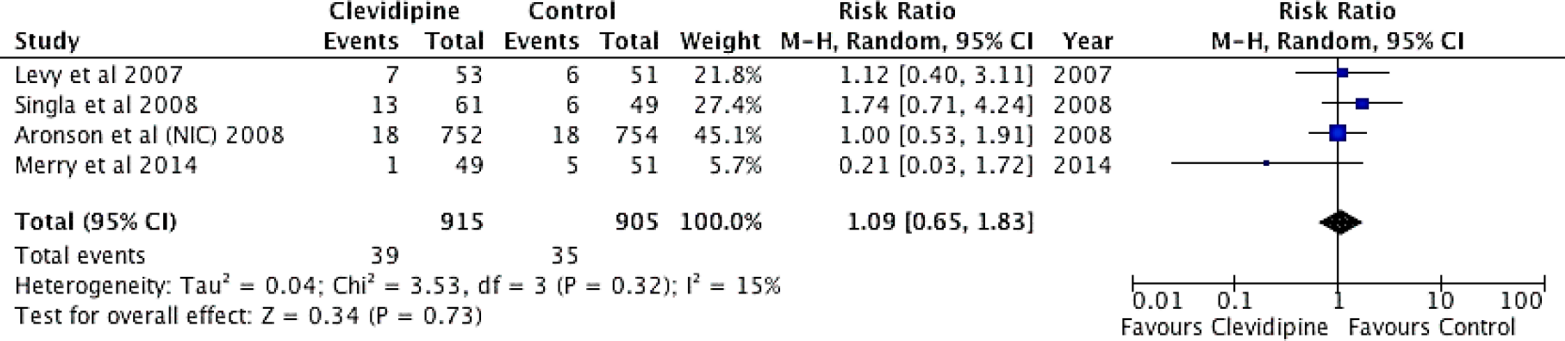
Forest Plot. Atrial Fibrillation. Forest plot considering p < 0.05 as statistically significant. One χ2 test was conducted for heterogeneity, considering p value of < 0.10 as statistically significant. NIC, denotes nicardipine.

No differences were found in the incidence of AF between clevidipine and control groups (RR 1.09, IC del 95%: 0.65 a 1.83; p = 0.73) (Fig 6).

### Publication bias

The insufficient number of RCTs included prevented the assessment of publication bias using the Funnel plot technique [16]. (Fig 2).

### Level of evidence (Fig 7)

**Fig 7:**
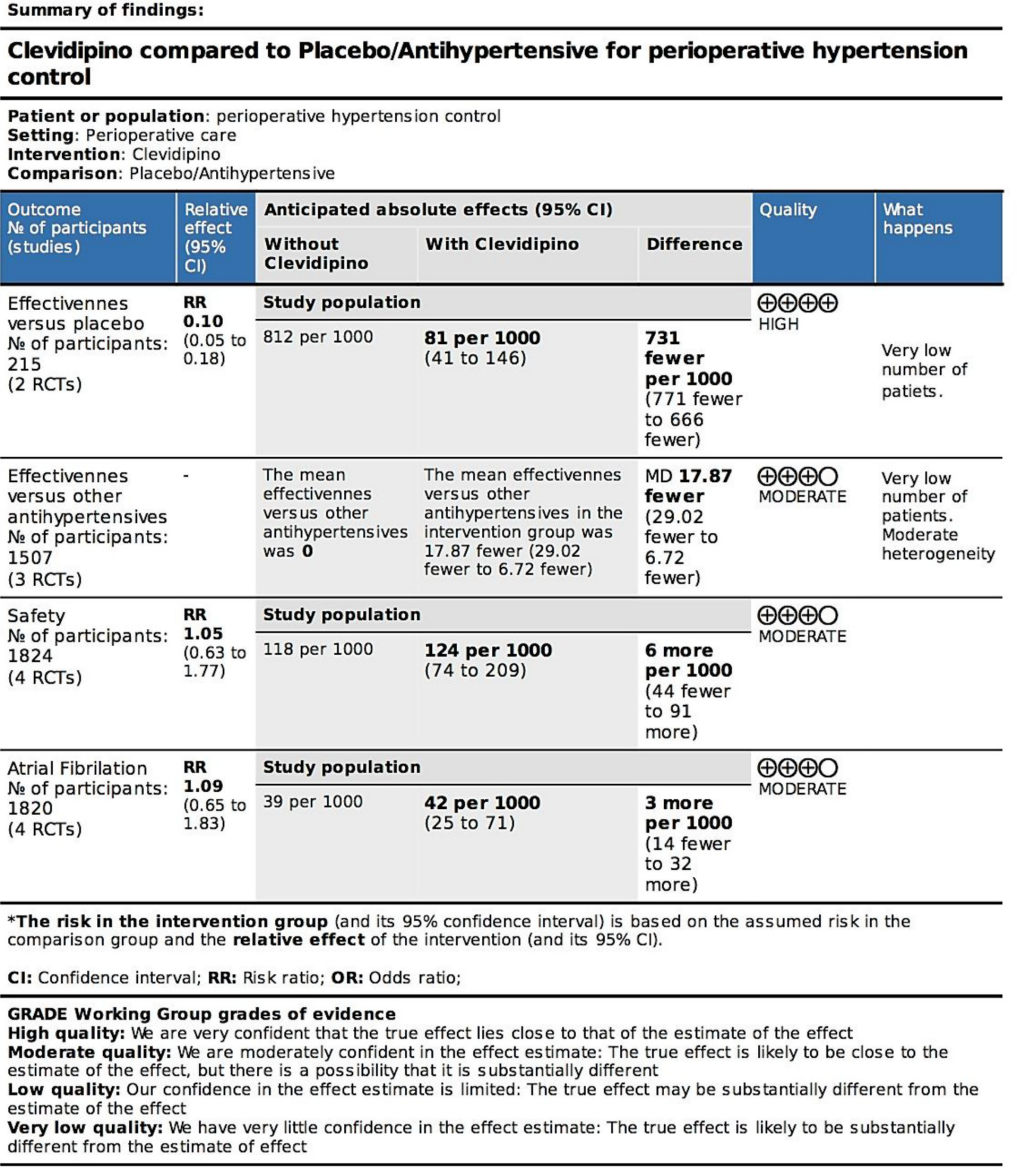
GRADE. GRADE summary of findings table.

## Discussion

To our knowledge, this is the first meta-analysis of the literature available for clevidipine. An optimal agent for perioperative blood pressure management should be a specific arterial vasodilator with rapid onset, short-term duration of effect, having low toxicity, and without the potential of causing reflex tachycardia [25].

Most vasodilators, such as nitroglycerin and sodium nitroprusside, act on both arterioles and venules and may cause undesirable reduction in cardiac preload. In addition, these drugs may impair renal and cerebral perfusion and induce intracranial hypertension. Nitroglycerine has an onset of effect of 2 to 5 minutes and duration of effect up to 20 minutes. Its administration is commonly associated with reflex tachycardia. Nitroprusside acts as arterial and venous dilator that can cause marked hypotension and can lead to cyanide toxicity [26, 27].

Beta-blockers, such as esmolol, decrease blood pressure, heart rate, and cardiac output and, therefore, they should be avoided in patients with bradycardia [28]. With labetalol, a selective alpha 1 and non-selective beta-blocker, cardiac output is maintained, and heart rate is modestly decreased or maintained. Labetalol has a rapid onset of action (2–5 minutes) and duration of action of 2–4 hours [6, 28].

Clevidipine has a rapid onset and duration of action, which allows its classification as an ultrashort-acting agent. The drug is manufactured as an emulsion of soybean oil and purified egg yolk phospholipids that make it lipophilic, with water solubility of 0.1 mg/m [6, 29]. The intravenous product is a mixture of two enantiomers S- and R-clevidipine [30]; each enantiomer has equipotent antihypertensive activity. At body temperature, the drug binds to plasma proteins (~99.7%) [31]. It is metabolized to inactive compounds by plasma and tissue esterases, with a mean depuration ratio of 0.121 Lit.min-1kg-1 [32] and a volume of distribution in steady-state of 0.6 L kg-1 [33]. Pharmacokinetic studies have shown a linear relationship between dosage and arterial blood concentration, achieving a steady state 2 minutes after the start of the intravenous perfusion [34]. Clevidipine does not depend on renal or hepatic function for its metabolism, and therefore, it has a superior safety profile compared to nicardipine (hepatic metabolism) and nimodipine (oxidative demethylation and dehydrogenation). Unlike the latter drugs, clevidipine can be safely used in patients with hepatic and renal disease. Clevidipine has been mostly studied in patients undergoing various surgeries, mostly cardiac procedures and to our knowledge clinical trials in neurosurgical patients have not yet been published [35].

Clevidipine reduces peripheral vascular resistance and, therefore, increases stroke volume and cardiac output. Its capability to selectively reduce the afterload prevents the influence over other hemodynamic parameters (increase left ventricle filling pressure and pulmonary wedge pressure) [8]. Its administration decreases systolic BP within the first 2–4 minutes after infusion [12], and baseline systolic BP and heart rate are achieved 15 minutes after the infusion is discontinued [36]. In contrast, nicardipine’s longer half-life results in a prolonged post-infusion effect [37]. In patients scheduled for CABG who required postoperative anti-hypertensive therapy to maintain MAP between 70–80 mmHg, clevidipine showed greater preload, stroke volume, and cardiac output. On the other hand, heart rate and systemic vascular resistance were lower and there were not significant differences in regional myocardial oxygen consumption or oxygen extraction, regional myocardial lactate extraction or uptake, and myocardial blood flow when compared to sodium nitroprusside. For a normotensive individual, clevidipine decreased significantly regional myocardial oxygen extraction during infusion. It also increased cardiac output and stroke volume by 10% without producing changes in the heart rate; coronary sinus blood flow increased 38% at the highest dose. [36, 38]

Two randomized, double-blind, placebo-controlled trials ESCAPE-1 and ESCAPE-2 demonstrated that clevidipine is appropriate and effective for the preoperative and postoperative BP management in hypertensive patients undergoing cardiac surgery [12, 13]. The reported incidence of treatment failure with clevidipine was 7.5% compared to 82.7% of placebo (per protocol rescue anti-hypertensive drug could be administrated after treatment failure). No treatment failure as a consequence of lack of efficacy was observed in the clevidipine group [12, 13]. In patients treated with clevidipine, median time to target BP (reduction of systolic blood pressure ≥15% from baseline) was 6 minutes in the ESCAPE-1 and 5.3 minutes in the ESCAPE-2 trials. The ECLIPSE trials involved analysis of three parallel comparisons, prospective, randomized, open-label studies, performed in 61 medical centers. In this trial, patients undergoing cardiac surgery were randomized in a 1:1 ratio to receive clevidipine or one of three antihypertensive medications (nitroglycerin, sodium nitroprusside, or nicardipine) [14]. Mean area under the systolic blood pressure time curve revealed that clevidipine was more effective than nitroglycerin or sodium nitroprusside, sustaining the BP in the specified range in the perioperative setting. Additionally, in the postoperative setting, there was not a significant difference between clevidipine and nicardipine (Fig 5). In general, clevidipine was well tolerated when administered during the perioperative setting in patients who underwent cardiac surgery [13, 39].

The decrease in BP was associated to an increase in heart rate in healthy volunteers treated with clevidipine [33], with a slight increase in heart rate also in hypertensive patients who received clevidipine in a moderate dose. A modest increase in heart rate, and not reflex tachycardia was observed in patients receiving clevidipine during cardiac surgery [12] or after coronary artery bypass grafting [38]. Clevidipine does not affect preload or venous capacitance, furthermore, as a dihydropyridine L-type calcium channel blocker clevidipine can produce a negative inotropic effect and potentially attenuate the reflex tachycardia triggered by its administration and rapid upward titration. Reflex tachycardia can be secondary to vasodilation and decrease in blood pressure. However, the effect on heart rate and the possible mechanism associated to reflex tachycardia remain to be elucidated. [29] As shown by Aronson et al., 30 days mortality in patients treated with nitroprusside was greater when compared to patients treated with clevidipine (4.7% vs. 1.7%, p = 0.04). However, no significant difference in mortality was observed at 30 days for stroke, myocardial infarction, and renal failure [14].

Other studies, not included in this meta-analysis, are equally conclusive, in 2003, Powroznyck et al. performed a randomized clinical trial in two medical centers in the United Kingdom comparing equivalent doses of clevidipine and nitroprusside [39]. In this study, they observed a greater heart rate increase with nitroprusside than with clevidipine (p<0.001); nitroprusside significantly reduced the systolic volume and central venous pressure and required greater intravenous fluid administration. Nitroprusside also exposed a greater incidence of hypotension as adverse event [39].

In this meta-analysis, we have included the double-blind study performed in four different centers by Merry et al. [40]. This study demonstrated no inferiority of clevidipine when compared to nitroglycerin. Although, the global incidence of adverse event was similar in both groups, arterial hypotension was more frequent in the group of patients treated with clevidipine (13 patients in clevidipine group vs. 8 in nitroglycerin group). Nevertheless, the double blinded (dummy) design of this trial was meant to be one of its strengths, but turned out to be a weakness, since it was more focused on the effectiveness of BP management rather that in its safety. One of the limitations of this study was the small sample size (45 and 48 patients in the clevidipine and nitroglycerin group respectively) [40].

An exploratory post hoc analysis of the ECLIPSE trials has detected an increased 30 days mortality associated with perioperative systolic BP variability in patients who underwent cardiac surgery [5].

Regarding the rate of atrial fibrillation the comparative study of clevidipine to nicardipine, sodium nitroprusside and nitroglycerin showed no difference in the incidence of this event in between treatments [14].

The ESCAPE-1, patients in the placebo and clevidipine group, experienced increases in heart rate from a baseline of 71 beats per minute (bpm) and 76 bpm, respectively, to a maximal heart rate of 84 bpm in both groups [12]. None of the patients included in the ESCAPE-1 trial withdraw the study medication due to lack of safety. In the ESCAPE-2 trial, there was no evidence of reflex tachycardia. However, atrial fibrillation was more frequent in the clevidipine group (21.3% vs. 12.2%), and this was the reason why clevidipine was withdrawn in one of the patients enrolled in the study [13].

In the ECLIPSE trials, comparator treatment groups and clevidipine were associated with similar rates of adverse events [14]. The most common adverse event was atrial fibrillation that was present in all the groups of treatment. Nonetheless, our analysis did not demonstrate an increase in atrial fibrillation. Only one serious adverse event (thrombophlebitis in a patient treated with clevidipine) reported in the ESCAPE-2 trial was considered to be associated to the study drug administration. Additional serious adverse events were reported as unrelated to clevidipine. In the ESCAPE-1 trial, a greater incidence of acute kidney injury has been reported in the patients treated with clevidipine when compared to the patients treated with placebo plus standard rescue antihypertensive intravenous drug (9% vs. 2%, respectively) [12].

From the total analysis completed over the 1,824 patients included in prospective studies, the incidence of adverse events was 94 in the clevidipine group vs. 107 in the control group; this shows a non-significant risk reduction of 1.05 (C.I 0.63–1.77). Clevidipine, therefore, displayed a similar safety profile regarding the studied adverse events when compared to other medications. Additionally, clevidipine is useful for the treatment of perioperative hypertension, due to the clinically effective outcome, as clevidipine did not present a greater number of adverse events when compared to placebo (+rescue antihypertensive intravenous drug) (Fig 3 and Fig 6). In conclusion, this meta-analysis supports the use of clevidipine in maintaining the blood pressure in a prespecified range ((Decrease in SBP >15% of baseline per ESCAPE 2 study) in the perioperative setting of cardiac surgery patients experiencing hypertension. Our study shows that clevidipine is effective and at least safe, when compared with other intravenous alternatives for perioperative hypertension management in patients ≥18 years-old undergoing on- or off-pump valve replacement or repair and/or CABG, or, minimally invasive CABG surgery. Our results do not provide evidence of clevidipine use during pregnancy, patients with cerebrovascular accident within 3 months before clevidipine administration, left bundle branch block, permanent ventricular pacing, intolerance to calcium channel blockers, allergy to the lipid vehicle of clevidipine. Clevidipine administration should be done according to manufactures instructions and titrations should be done according to clinical criteria [25].

### Limitations

There was moderate heterogeneity in the efficacy analysis in the group of studies that compare clevidipine with other antihypertensive agents (ECLIPSE), possibly due to the different characteristics of the comparators. Moreover, the number of patients and studies analyzed is very limited; and small studies tend to overestimate the effect.

Some studies could not be included as part of this meta-analysis due to methodological differences and other biases.

### Implications In Future Research

Two other studies have shown the effectiveness of clevidipine against other vasodilators, however, these studies did not meet inclusion criteria, and hence probably the degree of evidence may increase with inclusion. Given the existent inconsistency, further studies that evaluate different outcomes are required (2015). A prospective multicentric randomized clinical trial designed to evaluate the efficacy and safety of clevidipine in the management of hypertension in non-cardiac surgeries, in critical care patients or patients’ experiencing hypertensive crisis or hypertensive emergencies is essential.

## Conclusions

Clevidipine is an appropriate drug for the management of acute perioperative hypertension, unlike other intravenous infusions; clevidipine did not show adverse effects described with nitrites or tachyphylaxis. It has a short-acting effect; it is easy to titrate due to a linear dose-response and shows a rapid “wash-out" following a half-life of approximately 1 minute, which is an advantage over other calcium channel blockers.

Even though there is a wealth of data and the wide experience with other agents, clevidipine has several advantages that make it an ideal option for the perioperative use with a pharmacokinetic profile of rapid onset and short duration of action; efficacy data displayed limited excursions outside the desired BP range and lack of renal and hepatic metabolism.

## Supporting Information

S1 PRISMA Checklist (DOC)

## Abbreviations

SBP: systolic blood pressure
AUC: area under the curve
CABG: Coronary artery bypass grafting
SV: Systolic volume
RCT: Randomized clinical trial
AF: Atrial fibrillation
